# Hypertension and Cushing’s syndrome: hunt for the *red flag*

**DOI:** 10.1007/s40618-024-02453-9

**Published:** 2025-03-18

**Authors:** De Martino M.C., L. Canu, I. Bonaventura, C. Vitiello, C. Sparano, A. Cozzolino

**Affiliations:** 1 Dipartimento di Medicina Clinica e Chirurgia, Università degli Studi di Napoli Federico II, Naples, Italy; 2 Department of Experimental and Clinical Biomedical Sciences, University of Florence, Florence, Italy; 3 Department of Experimental Medicine, Sapienza University of Rome, Viale Regina Elena 324, Rome, 00161 Italy

**Keywords:** Secondary hypertension, Cushing’s syndrome, Hypercortisolism, Blood pressure, Glucocorticoids

## Abstract

**Introduction:**

The prevalence of secondary hypertension is reported to be 5–15% of people with hypertension. Causes of secondary hypertension include Cushing’s syndrome (CS), a rare but serious clinical condition characterized by chronic endogenous hypercortisolism associated with increased morbidity and mortality, especially for cardiovascular complications. The challenge for the clinician is thus to identify the phenotype of hypertensive patients who should be screened for endogenous hypercortisolism.

**Methods:**

This study was performed according to the PRISMA statement. The search was last updated in June 2023, and only English language studies were considered. Titles and abstracts have been screened for articles selection, identifying only those that dealt with prevalence of Cushing’s syndrome in hypertensive patients. Finally, eight papers were included in the review. Data regarding year of publication, populations’ characteristics, inclusion criteria, screening test and cut-off used, and CS prevalence have been extracted.

**Results:**

The study search identified eight studies, from 1977 to 2020, including a total number of 11,504 patients, ranging from 80 to 4429 patients for each study. The prevalence of CS reported was variable among the studies, ranging from 0 to 7.7%, having Cushing’s disease (CD) a prevalence range of 0-1.2%. The highest prevalence has been found in selected populations of hypertensive patients younger than 40 years (6.2%) or harbouring an adrenal lesion (7.7%). The most used screening test was 1 mg overnight dexamethasone suppression test (1 mg DST), with different cut-off.

**Conclusion:**

The most fitting CS profile encompasses younger age (i.e., < 40 years old), rapidly evolving hypertension and the presence of adrenal adenomas, along with subjects with pituitary lesions, who should still be prioritized in the diagnostic pathway. Overall, in the case of hypertensive patients presenting a clinical picture highly suggestive of CS, it is advisable to perform one of the available screening tests (UFC, 1 mg DST, LNSC). LNSC is likely the most discriminatory test and may be preferred, depending on its availability. Conversely, for hypertensive patients with an adrenal incidentaloma, the 1 mg DST is recommended as the screening test to exclude CS.

## Introduction

Elevated blood pressure secondary to an identifiable cause is defined as secondary hypertension [1]. The prevalence of secondary hypertension is reported to be 5–15% of people with hypertension [2, 3]. However, screening for secondary hypertension should be performed only in patients with specific characteristics that may suggest those with a higher likelihood of secondary cause, as screening all hypertensive patients is neither numerically feasible nor cost-effective. Secondary hypertension often presents with clinical findings that suggest its presence and even its specific aetiology. Endocrine causes of secondary hypertension include primary aldosteronism, which is the most common, while Cushing syndrome (CS) and phaeochromocytoma are observed less frequently [2]. Nevertheless, all these conditions should be considered in the diagnostic workup of patients with suspected endocrine hypertension. The present review will primarily focus on CS.

CS is a rare but serious clinical condition characterised by chronic endogenous hypercortisolism associated with increased morbidity and mortality, especially for cardiovascular complications [4, 5]. Endogenous CS is typically attributed to the excessive release of adrenocorticotrophic hormone (ACTH-dependent CS), primarily originating from a corticotroph pituitary adenoma known as Cushing’s disease (CD), which accounts for approximately 70% of cases. Less frequently, it can result from an extra-pituitary tumour secreting ACTH or, exceptionally rare, corticotropin-releasing hormone, leading to ectopic CS, responsible for about 10% of cases. Benign or malignant adrenal cortisol-secreting tumors (ACTH-independent CS) are accountable for approximately 20% of cases [6]. The estimated prevalence of CS is about 40 cases per million with an estimated incidence ranging from 0.7 to 2.4 cases per million per year [7–9]. According to epidemiological studies, the prevalence of hypertension in endogenous CS is around 80%, irrespective of sex and type (pituitary or adrenal CS) [10–13]. Data from the European Cushing’s Syndrome Registry showed that of all 466 patients examined, hypertension was one of the most common features at diagnosis (78%), especially in the ectopic CS, where it reached 88% [14]. These results confirm what was reported in a smaller cohort of patients with CS, in which the prevalence of hypertension was 85.1% [15]. Moreover, when considering age, the prevalence of hypertension has been observed to be higher in older patients (age ≥ 65 years), irrespective of sex, compared to younger (age < 65 years), as well as in comparison to patients of the same age with non-functioning pituitary adenomas. This suggests that the negative, multisystemic effect of hypercortisolism may outweigh those caused by aging alone [16, 17].

Hypertension is usually an early comorbidity of CS and may persist even after achieving disease remission [18, 19]. A non-dipping blood pressure profile, mirroring the disrupted cortisol circadian rhythm, is observed in more than 50% of patients with CS, whether normotensive or hypertensive, and in patients with adrenal hypercortisolism nocturnal decline is more blunted [20–22].

The pathophysiology of CS-related hypertension is multifactorial, with several mechanisms through which hypercortisolism impairs blood pressure, both directly and indirectly [10, 23].

Hypercortisolism enhances mineralocorticoid activity, since supraphysiological cortisol levels exceed the capacity of 11β-hydroxysteroid dehydrogenase type 2 (11β-HSD2). In a physiological condition, 11β-HSD2 effectively inactivates cortisol to cortisone in specific tissues where the mineralocorticoid receptor is expressed, in order to mitigate the negative effects of cortisol. This regulatory mechanism is compromised when the quantity of cortisol surpasses the enzyme’s capacity, resulting in cortisol binding to mineralocorticoid receptors and subsequent activation. This activation leads to sodium and water retention, an increase in plasma volume, and a simultaneous rise in potassium excretion, potentially causing hypokalemia, similarly to primary aldosteronism, being cortisol to cortisone ratio a marker of CS [24, 25]. The enhancement of mineralocorticoid activity plays a pivotal role in the distinct hypertensive phenotype observed in all the forms of CS but more frequent and severe in ectopic CS and in cortisol-secreting adrenocortical carcinoma [26–28].

Renin-angiotensin system is also impaired in CS. Indeed, cortisol induces an up-regulation of angiotensin II-receptors type 1, enhancing a vascular pressor response to angiotensin II.

Moreover, glucocorticoids modulate the synthesis and the vascular response to catecholamines. In patients with CS, an enhanced pressor response to beta-adrenergic agonists and impaired cardiac sympathetic autonomic modulation have been also demonstrated. In addition, hypercortisolism enhances vascular responsiveness to vasoconstrictors, leading to generalized vasoconstriction, which contributes to increased blood pressure levels.

Additionally, cortisol exerts an indirect effect on blood pressure and vascular system through CS systemic complications, being metabolic syndrome and obstructive sleep apnea also contributing to the pathogenesis of hypertension in patients with CS [10, 23].

Given the high prevalence of hypertension in these patients, an early diagnosis of CS in hypertensive patients would be advisable, aiming to reduce cardiovascular complications to which these patients are predisposed. The challenge for the clinician is thus to identify the phenotype of hypertensive patients who should be screened for endogenous hypercortisolism [29].

## Materials and methods

**Fig. 1:**
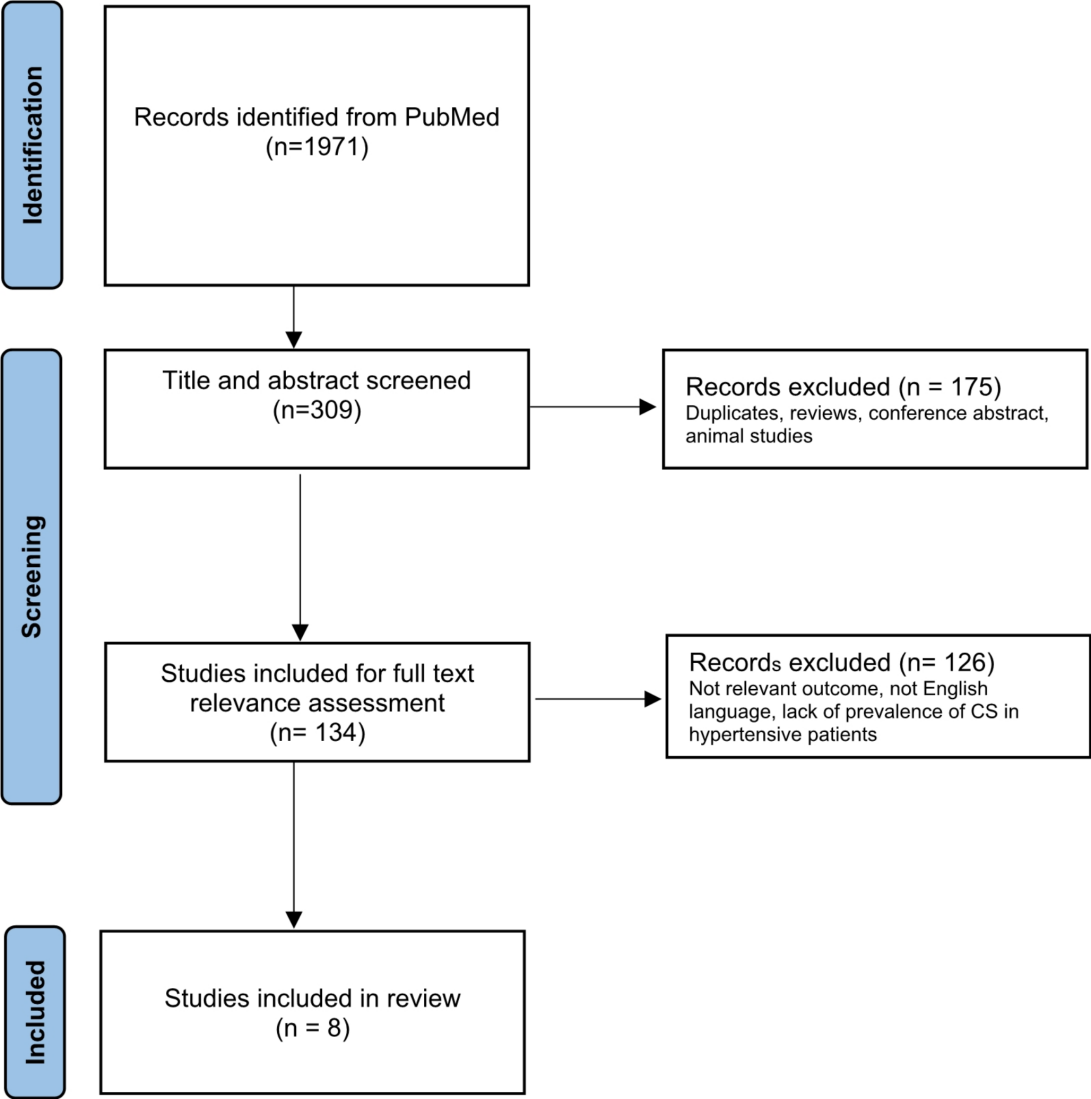
Flowchart of literature search on prevalence of Cushing’s syndrome in hypertensive patients (articles published in English from 1977 to 2020)

This study was performed according to the PRISMA statement [30]. In order to identify the pool of hypertensive patients who would merit screening for CS, we considered secondary hypertension prevalence studies on different types of hypertensive populations. We performed a keyword based PUBMED search, using relevant keywords [(hypertension[MeSH Terms]) OR (high blood pressure[MeSH Terms]) AND (Cushing’s Syndrome[MeSH Terms]) OR (Cushing’s Disease[MeSH Terms]). The search was last updated in June 2023, and only English language studies were considered. Titles and abstracts have been screened for articles selection, identifying only those that dealt with prevalence of CS in hypertensive patients. The selected abstracts were further assessed for a full-text evaluation. Finally, eight papers were included in the review. Data regarding year of publication, populations’ characteristics, inclusion criteria, screening test and cut off used and CS prevalence have been extracted. The flow diagram of the search strategy is displayed in Fig. 1.

### Prevalence of CS in patients with secondary hypertension

The study search identified eight studies, from 1977 to 2020, including a total number of 11,504 patients, ranging from 80 to 4429 patients for each study [31–38]. The prevalence of CS reported was variable among the studies, ranging from 0 to 7.7%, having Cushing’s disease (CD) a prevalence range of 0-1.2%. Variability is attributable to different hypertensive populations evaluated and different tests used for screening. Details of the included studies are summarized in Table 1.

**Table 1:** Details of studies evaluating prevalence of Cushing’s syndrome in hypertensive patients

Authors (ref)	Year	Country	*N*° of patients	Inclusion criteria	Test (cut-off)	Prevalence of overt CS (%)	Prevalence of MACS (%)
*Rudnick et al.* [36]	1977	Canada	665	Hypertensive patients aged ≥ 16 years [16–29 years: BP ≥ 140/90 mmHg; 30–69 years BP ≥ 160/100 mmHg; > 70 years BP ≥ 170/100 mmHg]	NA	0.2	NA
*Danielson et al.* [37]	1981	Sweden	1000	Hypertensive patients aged 20–70 years [< 40 years: BP ≥ 160/95 mmHg; 40–60 years: BP ≥ 170/105 mmHg; > 60 years: ≥180/100 mmHg]	Cortisol level (NA)	0.1	NA
*Sinclair et al.* [38]	1987	Scotland	3783	Hypertensive patients	NA	0.1	NA
*Anderson et al.* [32]	1994	USA	4429	Hypertensive patients aged > 17 years	Cortisol level (NA)	0.5	NA
*Omura et al.* [31]	2004	Japan	1020	Hypertensive patients	1 mg DST (> 3.0 µg/dL)	1.0 (CD 0.5; Adrenal CS 0.6)	1.0
*Martins et al.* [33]	2012	Brazil	423	Resistant hypertensive patients (using at least three antihypertensive drugs in full dosages) aged up to 80 years	LNSC performed twice (> 3.6 nmol/L)	0	NA
*Trifanescu et al.* [34]	2013	Romania	80	Hypertensive patients aged ≤ 40 years	1 mg DST (≥ 1.8 µg/dL)	6.2 (CD 1.2; Adrenal CS 5)	1.2
*Aoe et al.* [34]	2020	Japan	104	Hypertensive patients with adrenal incidentaloma	1 mg DST (≥ 5.0 µg/dL)	7.7	18.3

1 mg DST, serum cortisol level after 1 mg dexamethasone test; BP, blood pressure; CD, Cushing’s Disease; CS, Cushing’s Syndrome; LSNC, late-night salivary cortisol; MACS, Mild autonomous cortisol secretion in adrenal incidentalomas; NA, not available

Three of the included studies considered hypertension as the only inclusion criteria [31, 32, 38]. One study included hypertensive patients on at least three antihypertensive drugs in full dosages, always including a diuretic, and considered at least moderately adherent by a validated questionnaire [33]. Two additional studies included hypertensive patients in whom the diagnosis of hypertension was made considering different blood pressure (BP) thresholds, with lower BP levels considered diagnostic for younger patients, based on their age [36, 37]. Another study included only hypertensive patients younger than 40 years referred by cardiologists for endocrine hypertension screening [34] and another one included patients with adrenal tumors detected during secondary hypertension screening [35].

It was observed that the highest CS prevalence was found in two studies, specifically 7.7% in a population of 104 patients with adrenal tumors detected during secondary hypertension screening (including both patients who received hormonal evaluations before imaging and those who performed adrenal imaging before hormonal evaluations) [35] and 6.2% in 80 hypertensive patients younger than 40 years referred by cardiologists for endocrine hypertension screening [34], suggesting that the selection of a suitable population to be screened is mandatory in this setting.

The study by Aoe and coworkers aimed to compare the characteristics of patients with adrenal incidentalomas with those of patients with adrenal tumors detected during hypertension screening. Despite the authors reported a superimposable prevalence of CS between the two groups (8.0% vs. 7.7%; *p* = 0.92), it is noteworthy that both adrenal incidentaloma and hypertension are considered risk factors for hypercortisolism. Consequently, it would have been expected to observe a greater CS prevalence in subjects presenting with both conditions. Furthermore, it has been widely reported that the prevalence of hypertension is higher in patients harbouring adrenal incidentalomas compared to the general population [23].

With regards to tests used for screening, two older studies used baseline serum cortisol and identified a prevalence of endogenous hypercortisolism between 0.1% and 0.5% [32, 37]. It is noteworthy that serum cortisol is not a reliable screening test for CS, and this should be taken into account when interpreting the prevalence reported in the earlier studies from 1981 to 1994, respectively. Thus, it is possible that prevalence has been either underestimated or overestimated. Considering three studies that used the 1 mg - DST as a screening method for hypercortisolism with post-test cortisol cut off value > 1.8 µg/dl, > 3.0 µg/dl and > 5.0 µg/dl, the prevalence of CS ranged from 1.0 to 7.7% [31, 34, 35]. While it might have been expected that a lower cutoff would result in a higher frequency, it is important to note that making a direct comparison between these three studies is not appropriate, as they include different populations. Only one study examined a population of patients with multidrug-resistant hypertension assessing the presence of hypercortisolism by two salivary cortisol assays at 11 p.m., thus defining the absence of overt CS [33]. Screening methods were not described for two old studies, reporting a CS prevalence of 0.2% and 0.1%, respectively [36, 38]. The prevalence of CS as a secondary cause of hypertension, albeit as rare but not as negligible as that found in the aforementioned studies, hints that screening for CS should be considered in those hypertensive patients with features suggesting an increased likelihood of hypercortisolism.

As indicated by ESC/ESH guidelines, young onset, sudden development of severe or worsening hypertension, or multidrug-resistant hypertension are all features that should prompt screening for secondary hypertension. In particular, CS should be excluded in the presence of the combination of any of the above, especially if 24-h ambulatory BP profiles do not show nocturnal dipping, with other clinical features specific for hypercortisolism: easy bruising, facial plethora, proximal myopathy or proximal muscle weakness, wide violaceous striae, hypokalemia, diabetes mellitus, or osteoporosis in young male individuals and in hypertensive patients harbouring an adrenal mass [2, 3, 23]. Table 2 shows the main features of hypertensive patients who should be screened for CS.

**Table 2:** Main features of hypertensive patients who should be screened for Cushing’s syndrome

**Demographic characteristics**	– Young patients (< 40 years) – Onset of hypertension in childhood
**Hypertension phenotype**	– Resistant hypertension (at least three antihypertensive drugs in full dosages) – Acute worsening hypertension in patients with previously documented chronically stable hypertension – Non-dipping profile – Presence of extensive hypertension-mediated organ damage
**Radiological findings**	– Incidentally detected adrenal lesions – Incidentally detected pituitary lesions
**Clinical characteristics suggestive of hypercortisolism**	– Easy bruising, facial plethora, striae, central obesity, skin atrophy – Proximal myopathy or proximal muscle weakness – Hypokalemia – Diabetes mellitus – Osteoporosis at young age and fragility fractures

Multi-drug resistance is one of the peculiar features of hypertension associated with CS. The elevation in BP levels in CS is related to the chronic exposure to high circulating cortisol levels, and achieving biochemical normalization of hypercortisolism is the optimal approach for BP control in these patients [23]. Anyway, despite the lack of specific studies focusing on the treatment of hypertension in CS, it is advisable to adopt a therapeutic strategy that considers its pathophysiology [10, 23]. In line with this approach, considering the non-dipping BP profile, which reflects the disrupted cortisol rhythm, a more consistent dose of antihypertensive drugs administrated in the evening may be recommended.

The most recent consensus on CS diagnosis and management indicates three screening tests for assessing cortisol secretory status [39]:

1. *late-night salivary cortisol (LNSC)*, which is based on the assumption that patients with CS lose the normal circadian nadir of cortisol secretion and points out the abnormal circadian rhythm;
2. *1 mg DST*, which assays impaired glucocorticoid feedback. Indeed, in healthy individuals, a supraphysiologic dexamethasone dose inhibits vasopressin and ACTH secretion, thereby decreasing cortisol levels. A serum cortisol higher than the cut off of 1.8 µg/dl at 8:00 in the morning, after 1 mg of DST given between 23:00 and 00:00 of previous night indicates a pathological response;
3. *24 h UFC*, which suggests a possible increase in bioavailable cortisol and should be measured at least on two or three 24 h urine collections, to account for intra-patient variability.

One advantage of UFC over 1 mg DST test is that the overall cortisol production remains unaffected by changes in cortisol binding globulin (CBG), dexamethasone metabolism or patient adherence; however, it is important to note that UFC can exhibit random variability as high as 50%. For the interpretation of this test, it is also important considering sex, BMI, age, urinary volume, and sodium intake. Therefore, other screening tests such as LNSC might be preferred for patients with renal impairment or clinically significant polyuria, as well as for patients with mild hypercortisolism. This consideration becomes particularly important when selecting the most suitable screening test for hypertensive patients, as renal function is frequently affected in this population. On the other hand, the use of 1 mg DST also shows pitfalls, primarily related to dexamethasone metabolism and potential for interactions with certain antihypertensive drugs, as calcium channel blockers, which can affect CYP3A4 enzymes [40]. Conversely, LNSC exhibits lower accuracy and should not be the preferred choice for patients with disrupted circadian rhythms, such as night­shift workers, and as a rule it may require more than one sample for a reliable assessment.

In this setting, sensitivity of all tests is above 90%; the highest rates are seen with 1 mg DST and LNSC and the lowest with UFC. Specificity is somewhat lower, with LNSC the most specific and 1 mg DST and UFC the least [39, 41–45].

According to 2023 ESH guidelines, in a population of subjects with hypertension, screening for CS should be performed with one among 1 mg DST, 24 h UFC or LNSC, and in case of abnormal initial result of a first screening test, positivity of at least one of the remaining screening tests is required [2, 3].

In recent decades, improvements in imaging techniques and their wider application have led to an increased discovery of adrenal incidentalomas, about 2% in the adult population, reaching 10% in people older than 80 years [46]. Recent studies have shown that, regardless of the degree of hypercortisolism, patients with cortisol-secreting adrenal adenoma have an increased risk of morbidity and mortality, so that more recent guidelines have moved beyond the distinction between possible autonomous cortisol secretion (PACS) and autonomous cortisol secretion (ACS) and proposed the term “mild autonomous cortisol secretion” (MACS) [47–50]. The 2016 ESE guidelines for the management of adrenal incidentalomas introduced the term PACS for patients without overt CS and a serum cortisol post dexamethasone between 51 and 138 nmol/L. The term “autonomous cortisol secretion” was proposed instead for higher values (> 138 nmol/L) [51]. However, latest guidelines recommend that in patients without signs and symptoms of overt CS a post-dexamethasone serum cortisol concentration above 50 nmol/L (> 1.8 µg/dL) should be considered as MACS without any further stratification based on the degree of cortisol non-suppressibility [52].

Considering the eight studies identified in the literature, the prevalence of MACS was reported in only three of them and it exhibited variability across the studies, ranging from 1.0 to 18.3%. This variability can be attributed to the diverse populations of hypertensive individuals under investigation, which included both younger hypertensive patients and hypertensive patients with adrenal incidentalomas [31, 34, 35]. To the best of our knowledge, the largest published study with an extended follow-up duration showed increased all-cause mortality and a high prevalence of associated metabolic comorbidities (hypertension, type 2 diabetes, and dyslipidaemia) in patients with cortisol-secreting adrenal adenoma [50]. In this study, considering 3484 patients with adrenal incidentaloma, hypertension was the most frequent comorbidity at initial diagnosis (65.3%) and the prevalence increased as a continuum from non-functioning adenoma (NFA) to PACS and ACS patients. The high prevalence of hypertension in this population was also confirmed by previous studies including 28 to 887 adrenal incidentalomas, which confirmed that the prevalence ranged from 42 to 70% and the severity of hypertension was closely related to the degree of hypercortisolism [48, 53–56]. In a study of 210 patients with adrenal adenoma, those who had midnight serum cortisol > 5.4 µg/dl had higher systolic blood pressure than patients with normal cortisol levels, even when the analysis was adjusted for age, suggesting that midnight cortisol concentration could be a reliable test to select a subgroup of patients with adrenal adenoma with a cardiovascular risk profile [57]. Therefore, normalization of hypercortisolism should be the priority in the management of these patients, who may consequently benefit from adrenal surgery [58]. Indeed, it has been demonstrated that the use of antihypertensive drugs in patients with adrenocortical adenoma is reduced after adrenalectomy [59–61].

According to the most recent ESE-ENSAT guidelines, if a hypertensive subject also has an incidental adrenal lesion, the recommended screening test for hypercortisolism is the 1 mg DST test [47]. Notably, it is advisable to consider the exclusion of pheochromocytoma through the measurement of plasma free metanephrines or urinary fractionated metanephrines, especially in patients with adrenal lesions displaying atypical features not common of benign adenomas. Furthermore, in patients with unexplained hypokalemia, in addition to excluding CS, the aldosterone/renin ratio should be assessed to evaluate the possibility of primary aldosteronism [52].

## Conclusion

Although CS diagnosis is still challenging in real practice settings, its impact on the cardiovascular system is remarkable and hypertension may act as an early *red flag* [10, 13].

Any cortisol secretion significantly affects patients’ health regarding morbidity and mortality [50]. This awareness reflects the endeavours in optimizing hypercortisolism terminology, stressing the need to recognize also very mild hormonal secretion, hence the new definition of MACS [47, 50].

However, the highest frequency of arterial hypertension in the general population compels to narrow down the CS screening while preserving a wide and effective diagnostic skill for large sections of the population [2, 3]. Of note, the most fitting CS profile encompasses younger age (i.e., < 40 years old), rapidly evolving hypertension, multi-drug resistance, and the presence of adrenal adenomas, along with subjects with pituitary lesions, who should still be prioritized in the diagnostic pathway [2, 3, 23, 34, 35]. Moreover, in selected cases, the concomitant presence of hypokalemia could be suggestive of CS, especially ectopic CS or cortisol-secreting adrenocortical carcinoma [27].

On the other hand, several open issues about the diagnostic tests and thresholds still prevent the achievement of a univocal diagnostic policy within different specialistic settings, such as internal medicine, cardiological or endocrinological contexts [2, 3, 39, 47]. Indeed, 24 h UFC is still probably the one whose use is most widespread, even though it presents the lower specificity (however above 90%) and sensitivity [2, 3]. Instead, LNSC, the test with the greatest specificity and sensitivity, is still the least widely used [39].

In this light, further efforts are required to overcome the present diagnostic restraints, combining different specialists’ visions by establishing a shared and cross-sectional endocrinological imprinting in each potential medical framework.

Overall, in the case of hypertensive patients presenting a clinical picture highly suggestive of CS, it is advisable to perform one of available screening tests (UFC, 1 mg DST, LNSC). If the initial result of the first screening test is abnormal, positivity of at least one of the remaining is required. LNSC is likely the most discriminatory test and may be preferred, depending on its availability. Conversely, for hypertensive patients with an adrenal incidentaloma, the 1 mg DST is recommended as the screening test to exclude CS.

CS has many facets and arterial hypertension should be observed with suspicion and appropriately investigated in specific minority groups.

## Data Availability

Data sharing not applicable to this article as no datasets were generated or analysed during the current study.
